# Editorial: Modeling Disease Spread and Control

**DOI:** 10.3389/fvets.2017.00199

**Published:** 2017-11-21

**Authors:** Tariq Halasa, Salome Dürr

**Affiliations:** ^1^National Veterinary Institute, Technical University of Denmark, Kongens Lyngby, Denmark; ^2^Veterinary Public Health Institute, University of Bern, Bern, Switzerland

**Keywords:** model, disease spread, control, network analysis, decision support

**Editorial on the Research Topic**

**Modeling Disease Spread and Control**

## Introduction

Infectious diseases are a major burden for health (1) for both humans and animals and pose a constant economic challenge for the global economy (2, 3). Climate change, intensive global trade, emergence/reemergence of infectious agents and of antimicrobial resistance, combined with intensive livestock production systems make prevention and control of livestock infectious diseases a major global challenge. This intensifies the demand for tools to aid in better understanding of disease spread for cost-effective contingency planning and disease prevention and control.

Mathematical and simulation models have contributed to improve our understanding of the population dynamics of infectious diseases. In addition, they have provided decision makers with tools to aid in disease prevention and control based on scientific evidence (4–6). This research topic includes 10 scientific studies presenting different aspects and implementations of mathematical modeling for disease spread and control. The studies can be divided into: (1) model construction (two studies); (2) network analysis (two studies); (3) tools for decision makers (four studies); and (4) cost-effective control of endemic diseases (two studies).

## Model Construction

Constructing and describing a model of livestock production systems is challenging, as the systems are complex and may vary largely. It requires determining the most appropriate structure to use and the elements to include in the model. An integrated conceptual analysis is presented in this study availing a guideline for the construction of infectious disease process models and a comparison between the different modeling approaches (Mancy et al.). The authors discussed the different motivations for use of models in epidemiological research identifying key steps in model design and use and presented a conceptual framework for guiding model construction and comparison, depending on the modeled epidemiological systems.

The impact of indirect transmission of foot-and-mouth disease (FMD) *via* explicit modeling of virus persistence outside the host (in the environment) on the overall spread of the virus was examined using a stochastic individual-based model on the example of wild boar populations (Lange et al.). The authors compared a situation where there is transmission *via* direct and indirect contacts and a situation where transmission occurs only through direct contact. The results showed that the simplified, direct transmission model underestimates necessary sample size in surveillance plans by up to one order of magnitude, but overestimates the area put under control measures. Consequently, incorporation of indirect transmission mechanisms in epidemiological modeling is necessary.

## Network Analysis

Livestock industries are increasingly connected in ways that make control strategies based on local geographic boundaries or proximity unsuccessful. Long distance and complex patterns of movements complicate our understanding of how diseases spread, and how they should be controlled. The impact of changing the activity level of the German pig trade network on the probability of disease outbreaks, size, and duration of epidemics was studied (Lebl et al.). The results showed that small changes of the activity level of the network would have dramatic effects on the outcomes. These results are important because they indicate that the activity level of a trade network should be considered when simulating disease spread between pig herds, as it may influence the results significantly.

Exponential random graph modeling was used to reproduce, understand and predict pig trade networks in different European production systems (Relun et al.). The results showed that production system and farm characteristics—such as the geographical location, the production type, belonging to a pig company or housing system—were key drivers of pig trade. Statistics on local network configurations was necessary to capture the clustering observed in pig trade networks. This work provides approaches to simulate realistic pig trade networks that may be included in epidemic models.

## Tools for Decision Makers

Resources are limited and hence control strategies must be effective. Modeling offers a unique opportunity to evaluate control strategies and decision-making in the absence of an actual outbreak, as well as to estimate resource requirements that are needed for an appropriate response. A modeling study was carried out to identify characteristics measurable during the early phase of a FMD outbreak that might be useful predictors of epidemic outcomes, such as the total number of infected premises (IPs), outbreak duration, and the total area under control (AUC) at the end of the outbreak (Garner et al.). The results showed that these outcomes were associated with the number of IPs, the number of pending culls, the AUC, and the rate of disease spread at days 7, 14, and 21 following first detection, as well as cattle density around the index herd. These findings show that information available early in the outbreak can indicate its likely magnitude.

Simple semi-quantitative model-based decision tools are presented aiming to estimate the likelihood and the consequences of the ultimate size of an ongoing FMD epidemic, using simulated and actual outbreak data (Willeberg et al.). The results showed that the number of outbreaks at day 14 after FMD incursion is a useful predictor of the final epidemic size. In addition, the authors recommended that EU member states adopt simulation models as tools to aid decision-making, while ensuring that the output of such models is clearly understood by decision makers.

An iterative tool was developed with the aim of estimating the resources needed during an outbreak of FMD and identifying areas with limited resources that can delay the control of the disease (Boklund et al.). Outcomes of a simulation model of FMD spread were used to determine the daily required resources. The results showed that the number of needed personnel was predicted to peak within the first week. In addition, the time needed for surveillance visits was predicted to be the most influential factor for the required personnel.

The spread of a hypothetical outbreak of FMD in Sweden was studied and different control measures were simulated and evaluated (Dórea et al.). The results showed that the density of farms in the area where the epidemic started would have little impact on the time to control the outbreak. However, spread in high-density areas would require more surveillance resources, compared to areas of lower farm density. Based on these results, FMD outbreaks could be kept limited in Sweden using the EU standard control strategy and a national standstill of 3 days.

### Cost-Effective Control of Endemic Diseases

Endemic diseases cause large economic damage to livestock production, which requires constant evaluation of strategies to cost-effectively monitor, control, and prevent these diseases. A stochastic individual-based model simulating the spread and control of *Mycobacterium avium* subsp. *paratuberculosis* (MAP) within a dairy cattle herd was presented (Kirkeby et al.). The results showed that it was possible to eradicate MAP from a dairy cattle herd. Nevertheless, from an economic stand point, this was not attractive since the expenses for the control actions outweighed the benefits.

A comparison between two nationwide control strategies for footrot and a no intervention scenario with the current situation was conducted, to quantify their net economic effects (Zingg et al.). This was done by sequential application of a maximum entropy model, epidemiological simulation, and calculation of net economic effects using the net present value method. The results showed that a systematic Swiss-wide management program under the application of the recent PCR diagnostic test is the most recommendable strategy for a cost-effective control of footrot in Switzerland.

## Conclusion

The use of mathematical modeling to support decision-making is noticeably increasing, as its importance is progressively recognized by decision makers. The current research topic provides approaches, methods, and models that can support this evolution. It also provides useful tools to support decision-making for contingency planning and for the prevention and control of animal diseases on both the herd and the national level.

## Author Contributions

All authors listed have made a substantial, direct, and intellectual contribution to the work and approved it for publication.

## Conflict of Interest Statement

The authors declare that the research was conducted in the absence of any commercial or financial relationships that could be construed as a potential conflict of interest.
